# The origin of smiling, laughing, and crying: The defensive mimic theory

**DOI:** 10.1017/ehs.2022.5

**Published:** 2022-03-03

**Authors:** Michael S. A. Graziano

**Affiliations:** Department of Psychology and Neuroscience, Princeton University, Princeton, NJ 08544, USA

**Keywords:** Emotional expression, smile, laughter, crying, peripersonal space, startle reflex

## Abstract

Why do we leak lubricant from the eyes to solicit comfort from others? Why do we bare our teeth and crinkle our faces to express non-aggression? The defensive mimic theory proposes that a broad range of human emotional expressions evolved originally as exaggerated, temporally extended mimics of the fast, defensive reflexes that normally protect the body surface. Defensive reflexes are so important to survival that they cannot be safely suppressed; yet they also broadcast information about an animal's internal state, information that can potentially be exploited by other animals. Once others can observe and exploit an animal's defensive reflexes, it may be advantageous to the animal to run interference by creating mimic defensive actions, thereby manipulating the behaviour of others. Through this interaction over millions of years, many human emotional expressions may have evolved. Here, human social signals including smiling, laughing and crying, are compared component-by-component with the known, well-studied features of primate defensive reflexes. It is suggested that the defensive mimic theory can adequately account for the physical form of not all, but a large range of, human emotional expression.

## Graphical abstract

**Figure:**
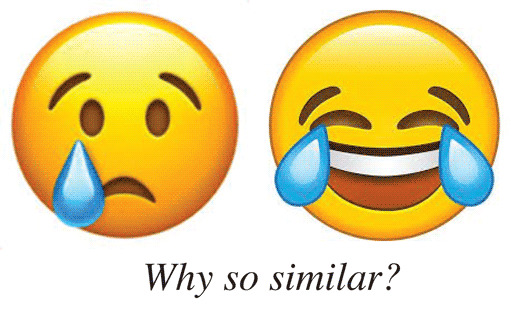

**Social media summary:** Smiling, laughing and crying may have evolved originally from defensive reflexes.

## Introduction

In a famous moment in Homer's *Odyssey*, a room full of villains laughs uncontrollably at a joke, their eyes streaming with tears. The poet evocatively paints the moment as foreshadowing the weeping they will soon be doing when Odysseus kills them all. Even 3000 years ago, observers of human nature noticed the eerie similarity between laughter and crying, especially as the intensity of the emotional expression increases.

Somewhat more recently than the Trojan War, my colleagues and I noticed that many human emotional expressions also resemble another domain of behaviour. Over many years we studied a set of reflexive behaviours in primates that protect the body surface – the startle reflex and other blocking and withdrawing reflexes (Graziano, 2018). These actions are so fast that they usually begin and sometimes end within a fraction of a second, but they can be studied using video recording and muscle activity measurements. On examining them in detail, we noticed that they resemble the suite of actions involved in smiling, laughing and crying (Graziano, 2008, 2018, 2021). Could defensive actions in some way have been an evolutionary starting point for many human emotional expressions? In this article, I will first summarise some of my laboratory's work on defensive reflexes, describing their physical characteristics. Next, I will outline the reasons why defensive reflexes might have been especially well suited to influencing the evolution of social signals. Finally, I will describe how smiling, laughing and crying might have an evolutionary origin in defensive actions. The overarching proposal that defensive movements are prone to shaping social signals, and that many familiar human social signals stem from that evolutionary origin, is here termed the ‘defensive mimic theory’. The term ‘theory’ is used in the sense of a unifying set of ideas that potentially explains a broad range of phenomena.

## Defensive actions

In this section, I will describe two kinds of defensive movements studied in humans and monkeys. The first is the startle response, which begins within milliseconds of a stimulus and depends on fast neural pathways that pass through the brain stem. The second is a more complex, long-lead defensive response that involves computing a margin of safety around the body, and depends on neural pathways through the cerebral cortex.

The first scientist to study the human startle reaction systematically was Strauss, working in the 1920s with the relatively new technology of a movie camera (Strauss, 1929). When he fired a pistol behind the heads of unsuspecting psychiatric patients (a procedure unlikely to pass a modern ethics review) and filmed the result, he observed a consistent set of component movements within the first fraction of a second, each component seemingly useful for protecting a part of the body (Davis, 1984; Koch, 1999; Landis & Hunt, 1939; Strauss, 1929). The musculature around the eyes contracted, causing a blink and a protective puckering of the skin. The musculature of the upper face contracted, causing the flesh on the cheeks to bunch upward, again protecting the eyes in folds of skin. An incidental consequence of this upward mobilisation of the cheeks was a characteristic lifting and curvature of the upper lip, causing the upper teeth to be exposed. The head pulled down and forward and the shoulders lifted, protecting the vulnerable neck. The torso curved forward, in Strauss's interpretation shortening the body and making it a smaller target for potential attack. The legs bent at the hips and knees, again making the body a smaller target for attack. The arms pulled in, bringing the hands into a central position guarding the abdomen, or sometimes in a higher position guarding the throat or face. Sometimes the sudden contraction of the torso forced air out of the lungs, which resulted in a sharp exhalation or a vocalisation.

The acoustic startle response is fast. The muscles around the eyes can begin to contract within 5 ms. This rapid onset is possible because the response is hard-wired into a short reflex arc from the auditory input to the brain stem (the pontine reticular formation), and from there to the muscle output (Davis, 1984; Koch, 1999). The response is also stereotyped. It does not protect varying parts of the body depending on the location or type of stimulus. Instead, it establishes a standard initial guarding posture from which a person can then produce a more situation-specific response using more complex reflexes.

Although the startle reflex does not itself require higher brain systems, the networks that process emotions and environmental context can evidently send signals to the brain stem and modify the overall gain on the startle reflex. As a result, the magnitude of the startle varies enormously depending on circumstances (Davis, 1984; Ehrlichman et al., 1995; Grillon, 2008; Grillon et al., 1991, 1996; Koch, 1999; Lang et al., 1990; McTeague & Lang, 2012). A calm person might have such a reduced response that only a slight tightening of the muscles around the eyes remains. A person in a state of stress or anxious anticipation will produce a larger startle response. As the magnitude of the reflex increases, it spreads from the eyes (where it is strongest) to other parts of the face, and then to other parts of the body.

After the initial startle reflex, a slower, more complex set of reflexes takes place. This second phase of the defensive reaction involves neural computations that construct a flexible, virtual margin of safety around the body, called peripersonal space. The system was first studied in macaque monkeys, and the findings were later confirmed in humans (de Vignemont & Iannetti, 2015; di Pellegrino & Làdavas, 2015; Duhamel et al., 1998; Graziano, 2018; Graziano et al., 1994; Rizzolatti et al., 1981). Neurons in specific, interconnected areas in the cerebral cortex respond to objects looming into the space near the body. Each peripersonal neuron is tuned to a restricted volume of space. For example, a peripersonal neuron may respond to a touch on the left cheek; to the sight of an object looming into the space within about 20 cm of the left cheek; and to nearby sound sources, with a preference near the left side of the face. The neuron therefore responds to a multisensory bubble of space attached to the skin surface. Anything that enters that bubble triggers the neuron. A range of neurons with different sizes of multisensory receptive fields, some hugging the body more closely and some extending outward farther, provides a map or representation of the space around the body. The representation extends from the skin to about a metre, with an emphasis on the space around the face and hands, and a greater representation of closer space with progressively less representation of farther space (Graziano, 2018).

Experiments in macaque monkeys showed that these peripersonal neurons play a special role in coordinating the second phase of defensive movements after the startle reflex. When electrical microstimulation was applied to areas of the cortex that contained peripersonal neurons, the stimulation evoked a set of movements consistent with defending a part of the body (Cooke & Graziano, 2004; Cooke et al., 2003; Graziano, 2015; Graziano et al., 2002). For example, consider a cluster of neurons that responds to a touch on the left cheek and to visual stimuli looming towards the left cheek. Stimulation of them will evoke the following suite of movements:

1. The eyelids close, most rapidly on the left.
2. the orbicularis muscle, which surrounds the eye, contracts, resulting in a protective squinting or puckering of skin around the eyes, with a stronger reaction on the left.
3. The nasolabialis muscle, running through the cheek, contracts, causing the skin on the side of the face to mobilise upward, pulling protective folds of skin up towards the eye. As a consequence, the upper lip pulls up, exposing the upper teeth. The exposure of the upper teeth differs mechanically from the exposure of teeth during chewing, biting or threat displays; during these other behaviours, the musculature surrounding the mouth contracts to withdraw the lips, whereas during the defensive reaction, the primary action is a lifting of the skin on the cheeks or snout, moving wrinkles towards the eyes.
4. The ears fold against the side of the head (macaque monkey ears are mobile), consistent with protecting the pinna from injury.
5. The head turns sharply to the right, consistent with avoiding a threat on the left.
6. The shoulders move upward rapidly, consistent with protecting the neck.
7. The left arm lifts, rapidly swinging the hand into a position that would shield the left side of the head.

These actions are consistent with a complex, spatially targeted reflex for protecting the left side of the face from an impending impact. A weak stimulation of the neurons will evoke a gentler version of the suite of actions, often manifesting only as muscular contraction around the left eye. A progressively stronger stimulation will evoke a stronger defensive response that extends across the face to the shoulder and arm.

For peripersonal neurons that represent the space near other parts of the body, microstimulation evokes actions appropriate for withdrawing or shielding those other body parts (Cooke & Graziano, 2004; Cooke et al., 2003; Graziano, 2015; Graziano et al., 2002). The movements have a latency as short as 30 ms, and present even in anaesthetised animals, suggesting that the neurons are close to a motoric output (matching their known connectional anatomy). The effects can be observed whether by electrical or chemical stimulation (Cooke & Graziano, 2004). Temporary deactivation of the peripersonal neurons results in a temporary reduction in defensive actions without reducing the activity of the same muscles in other behaviours (Cooke & Graziano, 2004). Therefore, an extremely broad range of results on the peripersonal network in the primate (human and macaque) brain has built up a now well-established picture of a complex, cortical reflex. Sensory information is combined along known pathways to construct a margin of safety around the body. When an object enters that margin of safety, the relevant neurons become active, their activity evokes a set of movements that protect the threatened body part and stronger activity evokes a stronger defensive response.

In summary, two main kinds of reflexes defend the body surface. The startle response, mediated subcortically, begins within 5 ms of the onset of a threat, and the peripersonal protective response, mediated by a cortical network of peripersonal neurons, begins within 30 ms. These two mechanisms operate together and represent the initial, involuntary response that protects the body.

## Defensive actions as data breaches

Defensive actions have three properties that make them uniquely relevant to the evolution of social signals (Graziano, 2021). First, they are easily visible to others. Second, they contain information about the internal state of an animal, especially about stress and anxiety. Third, they are important for survival and cannot be safely suppressed. These properties pertain both to the short-latency startle mechanism and the longer-latency peripersonal space mechanism.

For simplicity of explanation, here I will use a hypothetical Monkey A and Monkey B, but of course the interactions are likely to be more complex and will sometimes involve more than two individuals. Suppose Monkey A is attacked by Monkey B. Monkey A responds with a startle reflex followed by a more spatially specific, blocking and withdrawing reflex. The defensive reaction is visible to Monkey B. A weak response will appear as muscular tension and pursing of skin around the eyes. A stronger response will appear throughout the face. An even stronger response will include ducking or cringing, the head down, the shoulders up, the torso hunched.

Monkey B could, in principle, use those visual cues. Although the defensive response is a reflex, the volume knob on the reflex is influenced by the animal's state of stress and emotion, by recent threats and events, and by the animal's own perception of the risks it faces in the moment. A weak defensive response indicates that Monkey A is relatively stress free, in a state of low fear, probably of higher status in the troop hierarchy, in a moment of health and strength. The stronger the defensive reaction, the greater the likelihood that the animal has internal stress and fear, indicating weakness or lower hierarchical status or a history of losing fights. Monkey A is, in a sense, leaking actionable information about itself. The situation is primed for the evolution of a social signal.

There is a diversity of opinion on how signals evolve in animals. Information-based theories posit that signals evolve to transfer information from one agent to another (Font & Carazo, 2010; Seyfarth et al., 2010; Slocombe & Zuberbühler, 2007). Non-information-based theories posit that signals evolve because they have a direct effect on the behaviour of others (Dawkins & Krebs, 1978; Owren & Rendall, 1997; Rendall et al., 2009). Here I do not take a stance on signals in general. With respect to defensive behaviour in particular, in the following sections, I suggest two processes. First, adapting to respond to informative cues in the environment may be a useful way to think about the evolution of the receiver. Second, once the receiver has evolved to respond in a specific way in a specific circumstance, then a more direct effect is likely to drive the evolution of the sender and of the signal itself, which evolves to manipulate the behaviour of the receiver. The specific evolutionary processes will be clarified below as I discuss how defensive reactions may have given rise to three particular human social signals: smiling, laughing and crying.

## Smiling

A widely accepted explanation exists for the evolution of the smile, or the affiliative gesture called the ‘silent bared teeth display’, as originating from a subservient or submissive display (Beisner & McCowan, 2014; De Marco & Visalberghi, 2007; Preuschoft, 1992; Thierry et al., 1989; Von Hooff, 1962). Here I will summarise that explanation but with my own emphasis on the potential role of defensive reflexes.

Social signals are thought to evolve to allow the sender to manipulate the behaviour of the receiver (Dawkins & Krebs, 1978; Fridlund, 1994; Grafen & Johnstone, 1993; Godfray & Johnstone, 2000; Krebs & Dawkins, 1984; Morton, 2017; Schmidt & Cohn, 2001). In that interpretation, one can distinguish three steps. First, a stimulus exists in the environment. Second, the receiver evolves to respond to the stimulus. Third, the sender evolves to deploy or exaggerate that stimulus to manipulate the behaviour of the receiver.

Could smiling have evolved from defensive actions, following this pattern? Imagine two monkeys that belong to the same social group. Suppose Monkey B looms towards Monkey A as a challenge to hierarchical status, entering peripersonal space and triggering a defensive reaction. Suppose the reaction is large. By implication, A is of lower hierarchical status, stressed, less likely to fight, more likely to flee. This visible defensive behaviour plays the role of the initial stimulus from which a social signal can evolve.

Now suppose that evolution has shaped the receiver. Monkey B's neural systems are tuned to be sensitive to a useful environmental cue, A's visible defensive behaviour. What reaction to that cue would be advantageous to B? One can imagine situations in which B might take advantage of the sign of weakness to press the attack. However, within a social group, B is more likely to benefit from reducing or stopping the attack. That reaction is adaptive because there is no hierarchical status to be gained by fighting the lower status A, and B can save energy and risk. During the interaction, no explicit cognitive process occurs. Monkey B does not look at the defensive reaction of A, judge its magnitude and explicitly figure out, ‘I don't need to fight this wimp’. Rather, natural selection has shaped the neural systems of B to create a social reflex. The sight of a large defensive reaction in A on the approach of B automatically triggers a reduction in overt aggression of B. Thus far, there is no social signal – just an environmental cue to which B has evolved to respond. One could say that information about A, contained within A's defensive movements, was present in B's environment, and allowed for the evolution of a specific response mechanism in B to benefit B's survival. It is in this sense that information may be a useful way to think about the evolution of the receiver.

Now the evolution of the sender and of the signal itself can occur. If monkey A happens to produce a specific behaviour, a defensive set, and if B sees it, it will cause a specific effect on B, reducing B's aggression. The behaviour is like a string that A can pull to cause a change in its environment that is advantageous to it. Natural selection therefore equips A with a mechanism for generating that behaviour in a social context. Now, in addition to the original mechanisms for generating a defensive reaction when a physical object looms into peripersonal space and threatens to collide with the skin, Monkey A has a separate mechanism, a social mechanism, that generates a mimic defensive action that is exaggerated, more visible, lasts longer than a startle reflex or a peripersonal defensive response, and can be produced outside the context of a looming collision. Thus a social signal, which looks like a distorted, extended defensive reaction, has evolved. The signal itself did not evolve to transmit information from A to B; rather, it evolved because of the direct effect it has on the behaviour of B, reducing aggression. Presumably, the evolutionary process does not stop there. The sender's ability to produce the signal, the exact form of the signal and the receiver's ability to detect and respond to the signal must continue to co-evolve as an integrated system.

This social signal has been observed in a range of primates from various species of monkeys and apes to humans (Beisner & McCowan, 2014; De Marco & Visalberghi, 2007; Orlowska, et al., 2018; Preuschoft, 1992; Thierry et al., 1989; Von Hooff, 1962). The exact form is slightly different in different species. In humans, its epicentre is around the eyes. The Duchanne smile, or genuine smile, involves the contraction of the orbicularis muscle around the eye, in addition to the flashing of teeth (Duchenne, 1990). One might ask how a flash of teeth, an obvious threat, could have evolved into a signal of non-aggression. However, I suggest that the question is mistaken. The exposure of the teeth is fundamentally different between the non-aggression signal and an aggressive display. Exposing the teeth to bite or threaten is more associated with the musculature immediately surrounding the mouth. In the non-aggression display, the exposure of the upper teeth is more associated with a contraction of the nasolabialis muscle, zygomaticus major, and other muscles that lie towards the sides of the mouth and that lift the cheeks, bunching them upward, causing a wrinkling of skin under the eyes. Imagine walking from a dark room into painfully bright sun. You produce a reflexive reaction including a pursing of skin around the eyes, partial closure of the lids, bunching of the cheeks upward, and a consequent lifting of the upper lip and exposure of the upper teeth. Everyone is familiar with the involuntary sun grimace. It has nothing to do with making a threat and everything to do with protecting the eyes. The facial components of a strong, genuine smile resemble those defensive components.

The proposal is not that the human smile *is* a defensive cringe, or that a defensive cringe *evolved into* a smile. I am suggesting that a smile evolved as an exaggerated *mimic* of a defensive reaction, because of the aggression-reducing impact of that stimulus on the behaviour of others. In analogy, one would not say that twigs are stick insects, or that twigs evolved into stick insects. Rather, stick insects evolved to mimic twigs because of the impact of that stimulus on receivers (birds). In the present hypothesis, a smile is a mimic defensive reaction, slower and more sustained (and therefore more visible) than a real defensive reaction, and is typically emitted in a social context to reduce aggression.

## Laughter

Imagine the most intense, stomach-clutching, tear-streaming laughter. The skin wrinkles and puckers around the eyes. The eyes close. The muscles in the cheeks mobilise the skin upward, further hiding the eyes in puckered folds. As the cheeks bunch upward, the upper lip is lifted, exposing the teeth. The shoulders lift, the torso curls forward, the arms and hands curl around the abdomen. Tears secrete. A repeated huffing sound is produced, sometimes unvoiced and sometimes voiced. Laughter appears to be a loud, exaggerated, extended mimic of a defensive reaction. Even the tears match a reflexive defence of the eyes. Could laughter be explained by a similar evolutionary process as for the smile, mimicking defensive reflexes? Here I argue the case, while acknowledging that the proposal is speculative (Graziano 2018, 2021).

Ethologists have described a gesture, common among many mammals, called the open-mouth play face (Cordoni et al., 2016; Darwin, 1872; Henry & Herrero, 1974; Jolly, 1966; Palagi, 2008, 2009; Preuschoft, 1992; Ross et al., 2010; von Hooff, 1962). When mammals play, they gently bite, and an open mouth with covered teeth may have evolved into a signal to regulate the play. Great apes, like most primates, have an open-mouth play face (Darwin, 1872; Ross et al., 2010; von Hooff, 1962). In addition to the visual display, great apes add a sound. When a chimpanzee is tickled, it opens its mouth and makes a series of huffing sounds. Bonobos, gorillas and orangutans do the same. Darwin (1872) noted the similarity between ape huffing and human laughter, and that similarity has been studied more recently in quantitative detail (Kret et al., 2021; Ross et al., 2010). By implication, at least the vocalised part of human laughter may have first evolved in the common ancestor of apes and humans. Other scientists argue that an analogue of play laughter can even be found in rats, which emit high-frequency sounds as a part of their social interactions (Panksepp, 2007).

A variety of hypotheses have been proposed for the origin of laughter, focusing on social interaction, play and laughter's reward value (Gervais & Wilson, 2005; Provine, 2004; Scott et al., 2014; Wood et al, 2017). Two kinds of laughter are distinguished: spontaneous and volitional (Bryant & Aktipis, 2014). Spontaneous laughter is hypothesised to have evolved early, perhaps millions of years ago, and volitional laughter is hypothesised to be a cultural expansion of the original behaviour. Although the broad outlines of these evolutionary accounts are generally well accepted, they also have limitations. These accounts almost entirely focus on the vocal sound and do not address the many other physical actions that make up laughter. The argument that I will present here is not meant to contradict the previous accounts, but rather to add to them, addressing aspects of laughter that have not been previously explained. My argument here depends on recognising that human laughter is more than an open mouth and a huffing sound. It is an integrated, multicomponent behaviour that spans the face and other body parts. As the intensity of the display increases, more of its components emerge. Tension around the eyes emerges first. As the signal increases in intensity, one observes the opening of the mouth, the huffing sound, the muscular tension that lifts the cheeks towards the eyes and lifts the upper lip; then the components that engage other body parts emerge, such as lifting the shoulders, hunching the torso and a blocking posture of arms and hands over the abdomen or face. Tickle-evoked laughter is particularly interesting in the present discussion, because it is a reaction to an intrusion into peripersonal space and closely resembles the defensive reflexes triggered by peripersonal neurons (Graziano, 2018). Here I argue that tickle-evoked laugher evolved originally from defensive actions, as a signal to regulate play fighting, and that it then took on broader social roles.

Play fighting is common in mammals. It is beneficial because it hones coordination in general and fighting skills in specific. Ideally, the participant should learn to land successful blows or bites, but at the same time, should avoid harming the sparring partner, which would end a useful interaction. The goals might be better accomplished if play fighting were regulated by social signalling.

Imagine two human ancestors, primates A and B, play fighting. B tries to penetrate the defences of A to make contact with a vulnerable body part, such as the abdomen or neck, while A blocks and protects using a typical suite of defensive reflexes. Its torso curls, its arms move into blocking postures, its shoulders lift to protect the neck, its facial muscles contract to protect the eyes. As the attacking hand looms farther into A's peripersonal space, the defensive reaction becomes stronger. If B makes contact with A's skin, the peripersonal neurons fire at peak activity and trigger an intense defensive reaction. If B lands a blow near the eyes or nose, an autonomic reaction causes A's tear ducts to secrete lubricant, protecting the eyes.

This visible defensive reaction contains information. It signifies that B has won a moment in the fight. It also signifies that B has reached the threshold of harming A – a threshold that, for the benefit of both parties, it is better not to cross. In the present hypothesis, evolution, operating within that context, shaped a receiving mechanism in the brain to take advantage of the available cues. The stimulus – the multicomponent, strong defensive reaction – triggers B to pull back, keeping the play fight safe for A. At the same time, the stimulus also acts as a reward. It signals that B has engaged in a skillful attack that slipped past A's defences, and B should learn the move for the future. None of the process is cognitively explicit. B does not think to itself, ‘I don't want to hurt him; I better back off. And hey, I won a point!’ Instead, a social reflex has evolved; the defensive reaction in the context of the play fight evokes an automatic reaction in B.

Finally, according to the hypothesis, evolution can shape the sender of the signal. If a strong defensive reaction in A causes B to temporarily suspend the attack, then it is advantageous to A to deploy that behaviour to avoid injury. Therefore, a mechanism evolves that mimics the defensive reaction in an exaggerated manner, at the appropriate moment during the play fight, when B's attack is about to land or has just landed. As a result, over millions of years, a complex, integrated, social mechanism emerges. In that mechanism, during a play fight, when B wins a point and lands a touch on a vulnerable, heavily defended body part, A deploys a loud, extended, exaggerated mimic of a defensive reaction. The explanation provides a compelling account of the facial and postural components that accompany tickle-evoked laughter. Yet how does the voiced part of the signal fit? Squeezing air from the lungs is a normal part of a defensive reaction, as described above. That auditory component is exaggerated in laughter. One possibility is that a signal that is multimodal, in this case both visual and auditory, is more effective (Hebets & Papaj, 2005). Adding an acoustic component to the signal allows it to be received immediately, regardless of where B is looking, thereby ensuring that the play fight is regulated and injury is avoided.

In this speculation, B's reaction is not all-or-nothing, but depends on the intensity of A's signal. While A's laughter is gentle, B continues the play attack; as the laughter intensifies, B is signalled that it is approaching a desirable goal; when the laughter rises to a sharp peak, B pauses and pulls back for a moment. The laughter acts to moderate B's attack, but it also acts as a reward to B, reinforcing a successful play attack that has penetrated A's defences. If you have ever tickled a child, you may be familiar with the dynamic. You may also be familiar with the pattern that laughter is evoked most consistently by touching specific, vulnerable, heavily defended parts of the body. I argue that this hypothesised evolutionary process reasonably explains the feature-by-feature similarity between defensive reflexes and tickle-evoked laughter. The strength of the explanation lies in recognising that laughter is not just a vocal phenomenon but is accompanied by a highly specific set of facial and postural components, all of which make sense as an integrated whole.

Humans laugh in a range of contexts outside of play fights (Martin, 2007). How did tickle-evoked laughter evolve into other social forms? Here I will speculate freely. If the account thus far is correct, then laughter in play fighting took on a role as a reward. It rewards a successful move that slips past a play partner's defences. With that laughter signal, animal A has the power to dispense a reward to animal B. Reward plays a crucial role in animal behaviour. It reinforces the behaviour that occurred immediately before the reward, and thus shapes behavioural repertoire. In a social species, each animal having the ability to dispense a small, token reward to others at any time, thereby shaping the behaviour of others, may create extremely complex social dynamics. The role of laughter in social reward has been noted before (Scott et al., 2014; Wood et al., 2017).

For example, consider a standup comic. He tells jokes and the audience laughs. The laughs serve as reinforcers shaping the comic's behaviour. His act is honed through years of experience, testing material, keeping bits that get the most laughs and dropping those that do not. He is effectively a rat in an operant conditioning chamber, shaped by reward pellets to press a lever, do a spin and hop through a ring. Whatever contortions get the reward, that is what the comic learns to do. The interaction still retains an echo of the dynamics of a play fight. A joke is a fast emotional or cognitive jab that lands a punch (not coincidentally called a punch line). It must be unexpected. The surprise element, that misdirects and sneaks past the expectations of the audience, is key to the joke. And having made the joke, a good comic backs off for a moment, even if only for a few seconds, letting the punch line hang for several beats, and then begins the next joke. In humor, timing is everything. Why? In this hypothesis, it is because the brain mechanism that underlies humor evolved first in relation to a physical play fight, in which timing and misdirection and surprise are literally everything to success.

Human laughter is also sometimes used negatively (Martin, 2007). As the famous Mel Brooks quote goes, ‘Tragedy is when I cut my finger. Comedy is when you fall into an open sewer and die’. Why do people laugh at the discomfort of others, communicating contempt? It could be argued that the negative variant of laughter is a minor modification of the positive variant. Imagine person A and B in conflict. B wins – knocks A over, trips him in the mud. B wins a social reward: bystanders laugh. Yet to A, the same signal indicates failure; whatever A has done should be avoided in the future. Therefore, the mechanism evolves to distinguish two, contextually defined forms of the signal: the social reward for desired behaviour and the social punishment for undesirable behaviour.

Perhaps only a species with extreme social sophistication could handle such a complex signalling mechanism. Laughter is not uniform but a collection of signals, used in a variety of contexts, to produce a range of opposing effects on others. The signals themselves are nearly identical across situations. Only subtle shifts in tone, facial expression and contextual framing, distinguish one signal from another.

## Crying

The evolution of crying is less well studied than the evolution of smiling or laughing. The reason is that crying, unlike laughing, is apparently unique to humans (Gračanin et al., 2018; Vingerhoets, 2013). Non-human animals produce distress calls, whereas humans cry, making comparative evolutionary studies difficult. Scientific speculations on crying date back to Darwin (1872), and many evolutionary hypotheses have been proposed since (Andrew, 1963; Gračanin et al., 2018; Hasson, 2009; Murube, 2009; Neuman, 2007; Provine et al., 2009; Vingerhoets, 2013). There is a general consensus that crying acts as a social signal, that it evokes comfort or support from others, and that it is usually although not always associated with distress (Gračanin et al., 2018; Hasson, 2009; Hendriks & Vingerhoets, 2006; Hendriks et al., 2008; Lane, 2006; Provine et al., 2009; Vingerhoets, 2013). Almost all speculation, hypothesis and data, however, focus on one aspect of crying: the production of tears. I argue here that this blinkered focus on tear secretion misses crucial aspects of crying that are clues to its origin. It is like the blind man who feels the elephant's tail and cannot figure out what kind of creature it is. I argue that human crying, in its most intense form, includes secreting tears, contracting the musculature around the eyes, bunching the cheeks upward, lifting the upper lip, lowering the head, raising the shoulders, hunching the torso, pulling the arms into a blocking posture around the abdomen or face, and repeated vocalisations. Everyone who has had an involuntary fit of intense sobbing knows these components. The behaviour resembles a loud, exaggerated and sustained mimic of a defensive set. No other species, as far as I know, solicits comfort by mimicking the reflexes normally triggered by a vigorous blow to the face. The mystery of crying is not why we evolved a signal to solicit comfort, but why it evolved such a specific physical form.

Many proposals begin with infant crying (Darwin, 1872; Gračanin et al., 2018). Human infants, like infants of other species, produce a distress cry that solicits protection and comfort from adults. Unique to humans, after a few months of life, tear production during distress vocalisation begins, and the suite of actions that form crying remains in the repertoire of social signals throughout adulthood (Penbharkkul & Karelitz, 1962). The signal must have some adaptive value that begins in infancy and extends into adulthood.

One can imagine many ways in which the defensive suite of movements might give rise to a social signal that solicits comfort. Here I offer one speculation to make the point, but other scenarios may be just as possible. Consider two human ancestors, A and B, belonging to the same group. B attacks A in a dispute over food, personal space or another reason. After the attack, B comforts A. Other members of the troupe might also comfort A by grooming or touching. In bonobos, the comforting sometimes takes the form of makeup sex (Clay & de Waal, 2013; Furuichi, 2011). Underlying these instances of comforting is an initial aggression that threatens social amity. Because fights are inevitable, it is adaptive to have a mechanism for comforting the victim afterwards to repair amity.

Many species have unique methods of fighting. Sheep bash horns. Deer lock antlers. Giraffes bang their necks together. Humans ball their hands into a boney club and sock each other in the face. We may take fisticuffs for granted as a normal part of human aggression, but note that it is an idiosyncratic, species-typical mode of aggression, especially among males. Suppose B punches A in the face. All the usual defensive reflexes deploy. The skin puckers around the eyes. The cheeks and upper lip pull up hard, further wrinkling the skin protectively around the eyes. The head ducks, the shoulders rise, the arms pull across the torso or the face, the eyes water in an autonomic response.

Individual B, the aggressor, needs a mechanism for recognising when it should reduce aggression and offer comfort, to repair social amity. Others in the group also need a mechanism for recognising when to offer comfort to A. The defensive reaction of A offers a potential cue. It is highly visible and diagnostic. In this hypothesis, humans evolved to respond to that particular cue. When I see you enact an extreme defensive set, especially the kind triggered by a violent blow on the face, it evokes an instinctive reaction in me. I am more likely to reduce aggression and give comfort. My reaction helps preserve social amity, which is advantageous to me. Now we have at least one possible account of how the receiver mechanism evolved.

Once the receiver mechanism is present, the sender mechanism can evolve. If A mimics that type of defensive reaction, even if no attack has happened, it should be able to extract comfort from B, or from others in the group. A can exaggerate the signal, extending it over seconds or minutes, to increase its effect on B. If A makes that display, the signal will press buttons in B's brain and evoke comforting behaviour, or at least inhibit aggression. The evidence suggests that crying does serve as a signal to elicit comfort and emotional support (e.g. Lane, 2006; Hendriks et al., 2008). One set of studies suggests that female crying ‘acts as a signal to alert males during a conflict that they have overstepped boundaries’ (Lane, 2006).

In this account, crying is not itself a facial protective action. It is a mimic of the defensive suite of actions. It is exaggerated, extended in time, sometimes noisy and therefore more detectable by the receiver. The present proposal does not necessarily contradict previous speculations that focus exclusively on tears as a social signal. However, the defensive mimic theory explains the larger, integrated suite of behaviours, many of which emerge progressively more clearly as the signal rises in intensity. No evolutionary account of crying should ignore the integrated suite of actions. I am also not claiming that a narrow behaviour of human ancestors punching each other on the nose formed the sole source of the distress and bodily defence that then initiated the evolution of the signal. Other kinds of injury or threat might also be part of a defensive-mimic origin story. However, it is worth noting that the components of crying neatly match the defensive set for a punch in the face, and a punch in the face is a species-typical mode of aggression. Maybe that human form of violence was a significant contributor to the evolution of the signal that solicits a cessation of aggression and an offering of comfort.

Note that the proposal for crying is essentially the same as for laughter. Both involve fighting (play fighting or real fighting), defensive reactions and defensive-mimic signals that moderate aggression. In this account, laughing and crying may apply to different contexts, but they evolved in parallel, shaped by similar dynamics, explaining why they share a similar form. One of the strangest aspects of human social signalling is that crying and laughing are eerily similar, component by component – vocalisation, facial actions, head and body postures, and the production of tears. Their dynamics are different: in weak form, crying seems more focused on contraction of facial musculature and tear production, whereas laughing seems more focused on vocalisation. In strong form, as more of their components emerge, they become nearly identical. A person in an uncontrollable, stomach-clutching, eye-streaming fit of laughter can look like a person crying. Sometimes only context distinguishes. The defensive mimic theory neatly explains the similarity.

## How general is the defensive mimic theory?

Of course, not all social signals stem from defensive reflexes. Yet a large range of human emotional expressions may have been influenced by them. The reason, it is argued here, relates to the fundamental properties of defensive reflexes. They are visible to others, they contain information about the internal state of an animal and they cannot be safely suppressed. They are unavoidable data breaches, and therefore ripe for evolutionary processes that result in social signals. In this article I discussed three particular classes of signals – smiling, laughing and crying. However, the defensive mimic explanation may be more general than these three categories.

For example, when a person is anxious or of low hierarchical status compared with others nearby, the person may stand in a characteristic way that resembles the startle posture. We are all familiar with the common, servile cringe: the shoulders raised slightly around the neck, the arms and hands drawn in around the abdomen or chest, the torso curved forward slightly (Darwin, 1872; Ellyson & Dovidio, 1985; Hall et al., 2005). In contrast, when a person is confident or communicating high hierarchical status, the exact inversion of the defensive posture appears: the head is held upright, in particular the shoulders are down, exposing the neck, the back is straight, the chest is out, the arms are at the sides or even spread expansively. I argue here that the difference is *not* simply that the confident person is trying to look larger or the subservient person smaller. Instead, both postures function as effective signals because one resembles a defensive stance in many of its features and therefore is associated with high stress and low status, and the other inverts the defensive stance and therefore is associated with low stress and high status.

Greeting rituals vary across cultures, but often involve touching body parts that are normally heavily protected by defensive reflexes (Bell, 1997; Rossano, 2015). People touch faces or hands, possibly signalling a mutual non-aggression by showing that normally defended skin surfaces are intentionally exposed to the other person. At least we do not go to the extreme of grasping each other's genitalia, as in some species of baboons (Dal Pesco & Fischer, 2020).

One of the most fundamental features of human social interaction is personal space, the protective zone around the body into which we prefer not to let other people (Graziano, 2018; Hall, 1966; Hediger, 1955). Personal space reflects internal state. Anxiety increases it and confidence shrinks it. When compared with the cultural norm, those who push into a crowd and stand close to others are unconsciously signalling confidence. Those who stay near the edges of crowds or shrink away to maintain a greater distance from others are signalling anxiety and low confidence. Personal space is hypothesised to be a product of the peripersonal neurons, described at the start of this article, that coordinate defensive reflexes. Therefore, once again, defensive mechanisms profoundly shape human social signalling.

The purpose of this article is not to claim that all of human social behaviour derives from a primitive set of defensive reflexes. The purpose is to argue that defensive reflexes played a disproportionately large, hidden role in the evolution of human social signalling. I argue that its impact can be seen in smiling, laughing and crying, along with subtle variants and subcategories of each, and in a large range of other postural expressions and social signals.

Because the defensive mimic theory is relatively new and has not been extensively studied or compared across species, it is not clear (to me) how much it may apply beyond humans. Some of the defensive-related expressions discussed in this article can be found in other primate species, but it may be that defensive reflexes have had a comparatively larger influence on human social signalling. Here I offer one final speculation. Social signals in many species are obvious – bright colours, stereotyped actions or clearly distinguishable sounds. In humans, social signalling seems more often to involve signals that differ from each other subtly or by context. Language is a paradigmatic example, in which small sound differences convey enormous differences in meaning. Perhaps the greater signalling intelligence of humans helped unlock the advantages of mimic defensive signals, a domain of social interaction in which the subtlest shades, intensities and contexts of mimic defensive movements can signal that I'm friendly, I'm servile, you've screwed up and I'm deriding you, I'm suffering and need help, you won that play attack, or I acknowledge that you've proven your mental prowess with that clever witticism. Mimic defensive movements may have expanded in humans into a vast, largely unconscious, subtextual language.

## Transparency and reproducibility

This article contains no experimental data.
